# LATE ONSET PSYCHOSIS AND VERY LATE ONSET PSYCHOSIS: WHAT ARE THE POSSIBLE ETHIOLOGIES?

**DOI:** 10.1192/j.eurpsy.2023.2203

**Published:** 2023-07-19

**Authors:** A. C. Ramos, S. C. Martins, T. M. Afonso, N. B. Santos, P. Gonçalves, T. Maia

**Affiliations:** Psychiatry and Mental Health Department, Professor Doutor Fernando Fonseca Hospital, Amadora, Portugal

## Abstract

**Introduction:**

Psychotic symptoms have long been known to show up earlier in life, typically during adolescence and early adulthood. Late Onset Psychosis (LOP), in which symptoms start between 40 and 60 years of age, and Very Late Onset Psychosis (VLOP), in which onset of symptoms happens after 60 years of age, although classically rare, have had a growing prevalence in the last decades.

**Objectives:**

To access the definition and main etiologies of LOP and VLOP, based on the current literature.

**Methods:**

Non-systematic review of literature using the terms “late onset psychosis” and “very late onset psychosis”. Case report of a patient who was admitted and treated in our inward patient field.

**Results:**

51-year-old female patient. She is divorced (two previous marriages) and has two daughters (26 and 16, respectively). She was brought by police officers because of behavior problems at the shelter where she was living. She was evicted from the house she was living in because of delay in paying the rent. On observation, she verbalizes persecutory and prejudicial delusions and auditory hallucinations on the 2^nd^ and 3^rd^ person (commenting voices) with at least 5 years of duration. She was hospitalized for almost 3 months, with slow but progressive clinical improvement on haloperidol 7,5mg/day. At the date of discharge, she did not spontaneously verbalize her symptoms, although she did not recognize them as delusional. Recent studies have shown that the prevalence of Schizophrenia in the typical age range is 75-80%, which means that an important proportion of diagnosis is made after that age span. Primary causes of LOP and VLOP are schizophrenia (of late onset), schizophrenia-like very late onset psychosis, delusion disorder, unipolar depression with psychotic symptoms and bipolar disorder. Secondary causes should also be considered, such as delirium, dementia (Alzheimer’s, Lewi bodies and vascular), and substances abuse; even more rare, other conditions should be considered, as cerebrovascular accident, encephalitis, epilepsy, and multiple sclerosis.

**Conclusions:**

LOP and VLOP have been a growing diagnosis in the past decades. In the assessment of these patients, we must consider the importance of secondary etiologies besides the primary psychiatric ones. Primary psychosis is a diagnosis of exclusion, and the clinician must rule out secondary causes. Recent data point out these symptoms as markers for an increased risk of dementia in these patients. Further research involving individuals with LOP and VLOPs is required to increase the evidence base for treatment and improve outcomes of care.

**Disclosure of Interest:**

None Declared

